# Dataset of breast ultrasound images

**DOI:** 10.1016/j.dib.2019.104863

**Published:** 2019-11-21

**Authors:** Walid Al-Dhabyani, Mohammed Gomaa, Hussien Khaled, Aly Fahmy

**Affiliations:** a Faculty of Computer and Artificial Intelligence, Cairo University, Egypt; b National Cancer Institute, Cairo University, Egypt

**Keywords:** Ultrasound, Breast cancer, Medical images, Dataset, Deep learning, Classification, Segmentation, Detection

## Abstract

Breast cancer is one of the most common causes of death among women worldwide. Early detection helps in reducing the number of early deaths. The data presented in this article reviews the medical images of breast cancer using ultrasound scan. Breast Ultrasound Dataset is categorized into three classes: normal, benign, and malignant images. Breast ultrasound images can produce great results in classification, detection, and segmentation of breast cancer when combined with machine learning.

**Table:** Specifications Table

Subject area	*Medicine and Dentistry*
More specific subject area	*Radiology and Imaging*
Type of data	*Images and mask images*
How data was acquired	*LOGIQ E9 ultrasound and LOGIQ E9 Agile ultrasound system*
Data format	*PNG*
Experimental factors	*All images are classified as normal, benign and malignant*
Experimental features	*When medical images are used for training deep learning models, they provide fast and accurate results in classification, detection, and segmentation of breast cancer.*
Data source location	*Baheya Hospital for Early Detection & Treatment of Women's Cancer, Cairo, Egypt.*
Data accessibility	https://scholar.cu.edu.eg/?q=afahmy/pages/dataset
Related research article	1. Walid Al-Dhabyani, Mohammed Gomaa, Hussien Khaled and Aly Fahmy, **Deep Learning Approaches for Data Augmentation and Classification of Breast Masses using Ultrasound Images** [1]

**Table:** 

**Value of the Data** • Ultrasound scan is mostly used for examination and early detection of breast cancer. Moreover, it is safe in comparison to other radiology imaging techniques. • Breast Ultrasound dataset can be used to train machine learning models which can classify, detect and segment early signs of masses or micro-calcification in breast cancer. • Researchers with interest in classification, detection, and segmentation of breast cancer can utilize this data of breast ultrasound images, combine it with others' datasets, and analyze them for further insights. • The data is comprehensive, containing breast cancer states (normal, benign, and malignant). • This dataset is – to our best knowledge – the first breast ultrasound dataset publically available.

## 1 Data

**Fig. 1:**
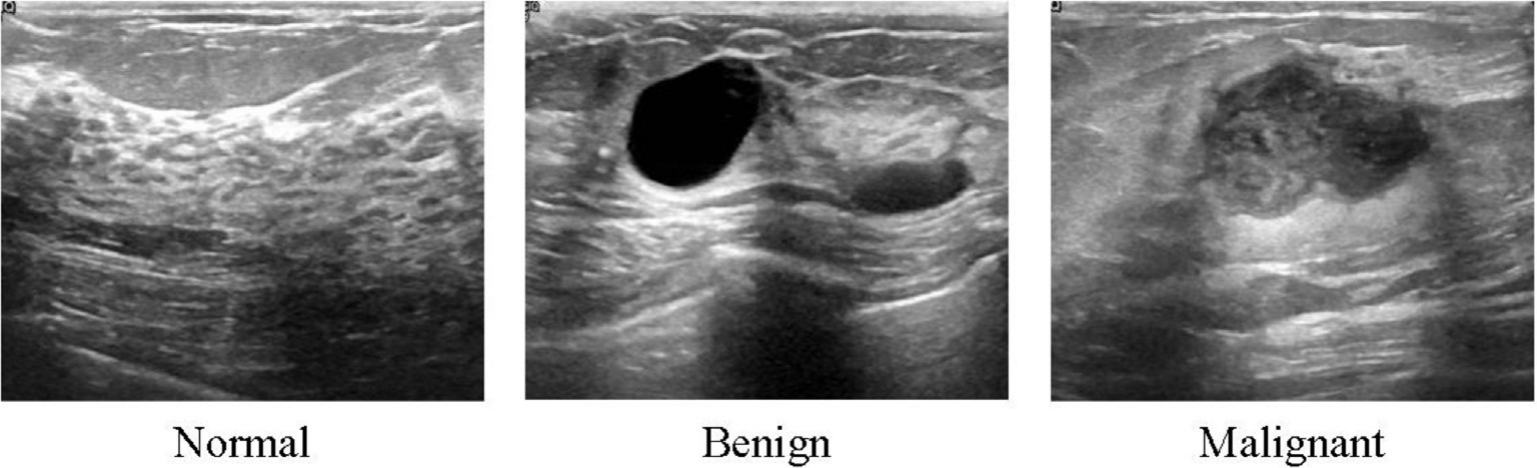
Samples of Ultrasound breast images dataset.

**Fig. 2:**
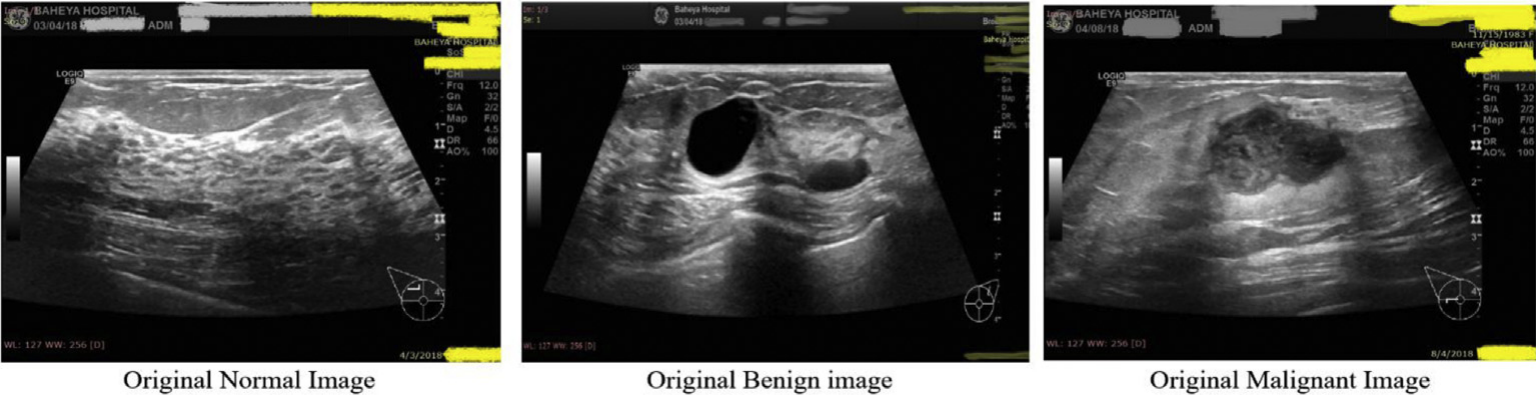
Samples of original Ultrasound breast images dataset (Original images that are scanned by the LOGIQ E9 ultrasound system).

**Fig. 3:**
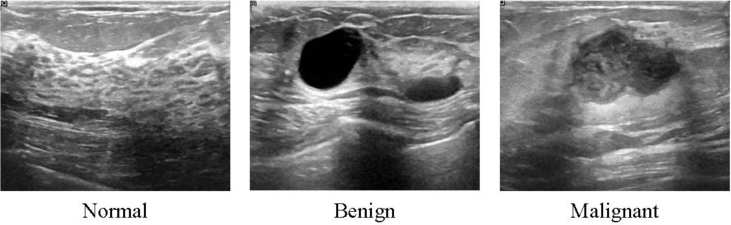
Samples of Ultrasound breast images dataset after refining.

**Fig. 4:**
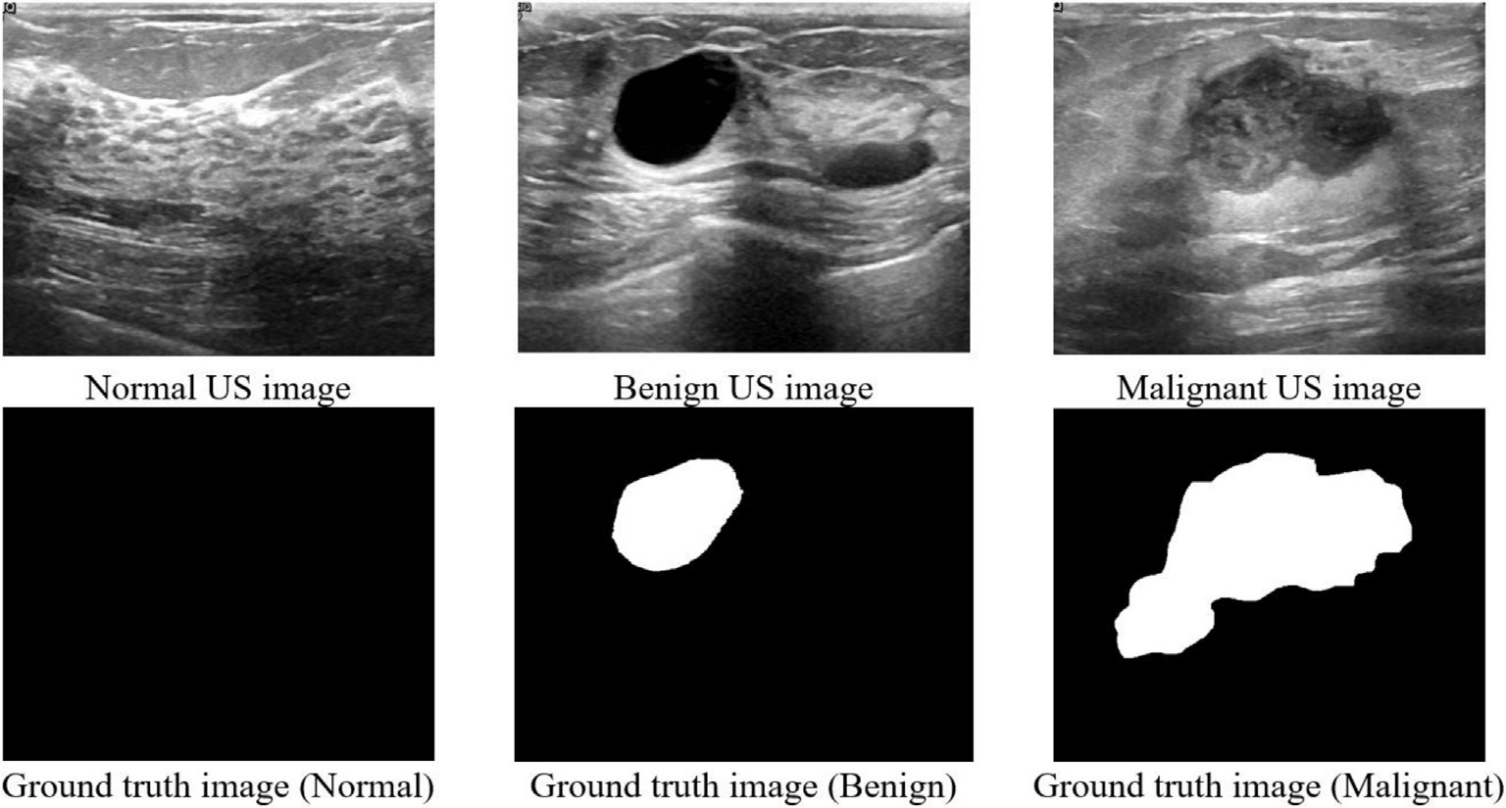
Samples of Ultrasound breast images and Ground Truth Images.

The data collected at baseline include breast ultrasound images among women in ages between 25 and 75 years old. This data was collected in 2018. The number of patients is 600 female patients. The dataset consists of 780 images with an average image size of 500 × 500 pixels. The images are in PNG format. The images are categorized into three classes, which are normal, benign, and malignant. The number of images in each class is shown in Table 1. The data samples are illustrated in Fig. 1. Samples of original images and the images after preprocessing are shown in Fig. 2 and Fig. 3, respectively. Furthermore, each image has its own ground truth (mask image) as shown in Fig. 4.

**Table 1:** The three classes of breast cases and the number of images in each case.

Case	Number of images
Benign	487
Malignant	210
Normal	133
Total	780

## 2 Experimental design, materials, and methods

### 2.1 Dataset collection

Ultrasound (US) images are generally in grayscale. They were collected and stored in a DICOM format at Baheya hospital. The consumed time used to collect and annotate the images is about one year. US dataset is categorized into three classes: normal, benign, and malignant. At the beginning, the number of images collected was 1100. After performing preprocessing to the dataset, the number of images was reduced to 780 images. The original images contain unimportant information not used for mass classification. Moreover, they may affect the output results of the training process. The instruments used in the scanning process are LOGIQ E9 ultrasound system and LOGIQ E9 Agile ultrasound system. These instruments are usually used in top-notch imaging for radiology, cardiac and vascular application. They produce image resolution of 1280*1024. The transducers are 1–5 MHz on ML6-15-D Matrix linear probe. Fig. 2 Illustrates a sample of the original scanned images.

### 2.2 Preprocessing

To make the dataset useful, some tasks should be performed. The data included duplicated images that required to be removed. Furthermore, radiologists from Baheya reviewed and fixed the incorrect annotation. DICOM images were converted to PNG format by using a DICOM converter application [2]. After refining the dataset, the number of US images was reduced to 780 images. The images are categorized into three classes (cases), which are normal, benign, and malignant. All images were cropped to different sizes to remove unused and unimportant boundaries from the images. We used fast photo crop [3] for this task. The image annotation is added to the image name. Special radiologists at Baheya hospital reviewed and checked all images. An example of the refined images is shown in Fig. 3.

### 2.3 Ground truth

Ground truth (image boundary) is performed to make the ultrasound dataset beneficial. Matlab [4] is used to perform this step. A freehand segmentation is established for each image separately. An example of mask images is shown in Fig. 4. Three folders are created for each type of breast cancer categories. Each folder has the images of its class. The image name includes the name of the class and the number of the image. Furthermore, the name of the masked image has the name as the US images with adding “_mask” to the end name of the image.

### 2.4 Ethical considerations

Researchers are mindful of the fact that patients have a right to be protected from public scrutiny of their private lives and illness. To this end, the researcher ensured that the patients and the hospital were adequately informed about the objective of this study. In addition, every patient's data stays unknown and his or her illness states is with the utmost confidentiality.

## References

[1] Al-Dhabyani Walid, Gomaa Mohammed, Khaled Hussien, Aly Fahmy (2019). “Deep learning approaches for data augmentation and classification of breast masses using ultrasound images”. Int. J. Adv. Comput. Sci. Appl..

[2] Medixant (2018). RadiAnt DICOM Viewer. https://www.radiantviewer.com.

[3] Śmieszny (2013). Fast Photo Crop. https://www.microsoft.com/ar-eg/p/fast-photo-crop/9wzdncrdnvpv?activetab=pivot%3Aoverviewtab.

[4] (2015). MATLAB and Statistics Toolbox Release.

