# Supplementary material for: Dataset of breast ultrasound images
Source: Data Brief. 2019 Nov 21;28:104863. doi: 10.1016/j.dib.2019.104863 (PMC6906728; doi:10.1016/j.dib.2019.104863)
Supplement: Multimedia component 1 [file mmc1.zip › Ethical Committee V2.pdf]

2.October.2019

Mr. Walid Saleh Mohsen Al-Dhabyani

Dear Walid

The Research Ethics Committee has recently reviewed your responses to the conditions placed upon the ethical approval for the project outlined below. Your proposal Is now deemed to meet the requirements of the National Statement on ethical Conduct in human Research (2007) and full ethical approval has been granted.

Approval No.	**Cairo 5.02.2018**
Project Title	A Machine Learning Approach for Diagnosing Medical Images of Breast Cancer
Approval date	1 February 2018
Expiry date	30 June 2020
BREC Decision	**Approved**

The standard conditions of this approval are:

(a) conduct the project strictly in accordance with the proposal submitted and granted ethics approval, including any amendments made to the proposal required by the BEC.

(b) Consent of patients for publication was waived by institutional committee approval.

(c) advise (email: noha.abdelrazek@baheya.org) Immediately of any complaints or other issues in relation to the project which may warrant review of the ethical approval of the project.

(d) make submission for approval of amendments to the approved project before implementing such changes.

(e) provide a 'progress report' for every year of approval.

(f) provide a 'final report' when the project Is complete.

(g) advise in writing if the project has been discontinued.

Please note that failure to comply with the conditions of approval and the National Statement (2007) may result in withdrawal of approval for the project.

Kind Regards,

REC CHAIRMAN

Dr. Amany Helel

Professor of Medical Oncology

Baheya Ethics Committee

Baheya Hospital

Signature: A. Helel

Date: 2. Oct. 2019
