# Supplementary figures and images for: Dataset of breast ultrasound images
Source: Data Brief. 2019 Nov 21;28:104863. doi: 10.1016/j.dib.2019.104863 (PMC6906728; doi:10.1016/j.dib.2019.104863)

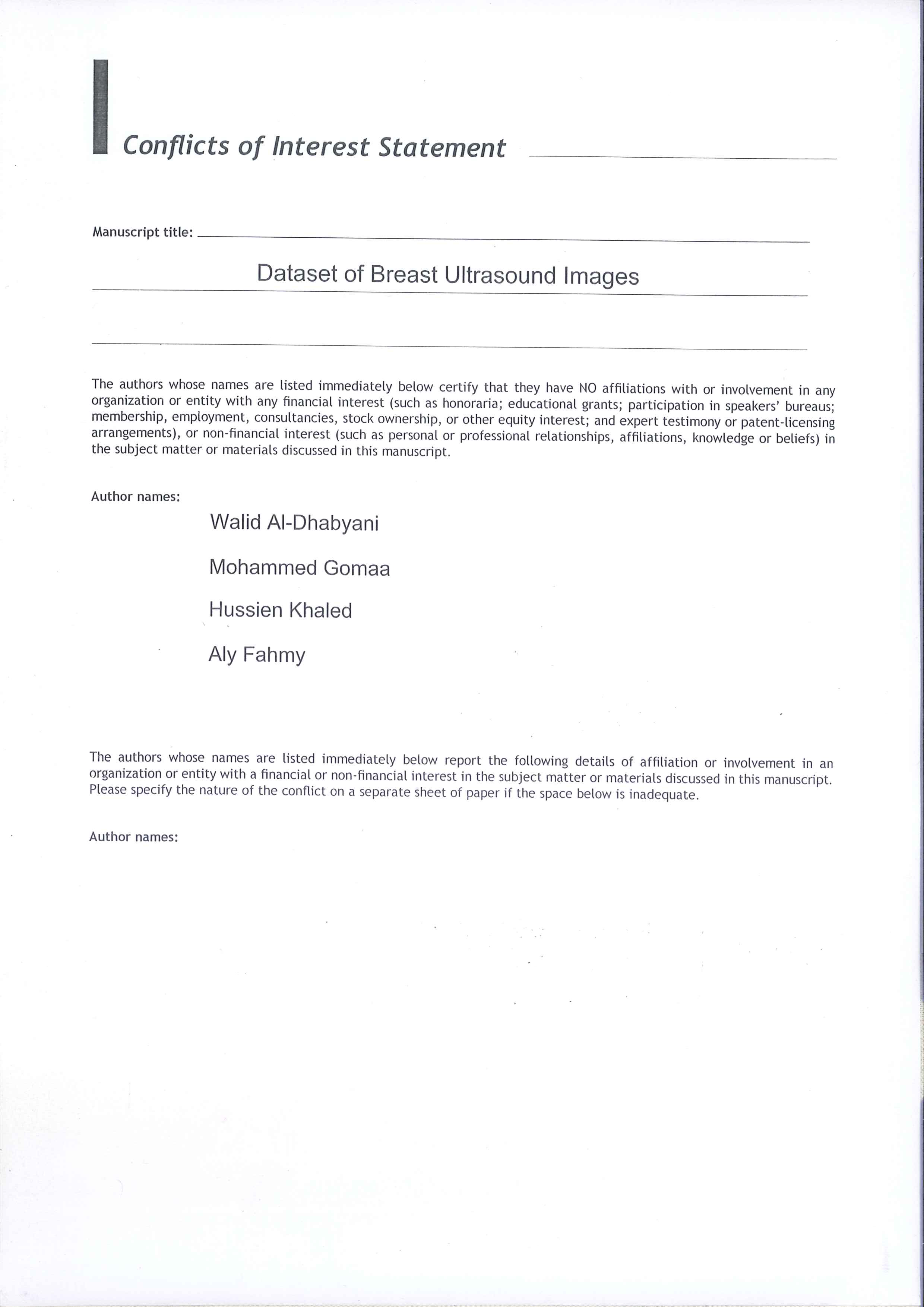

Supplement: Multimedia component 1 [file mmc1.zip › confilct_of_interset_statement_1.png]

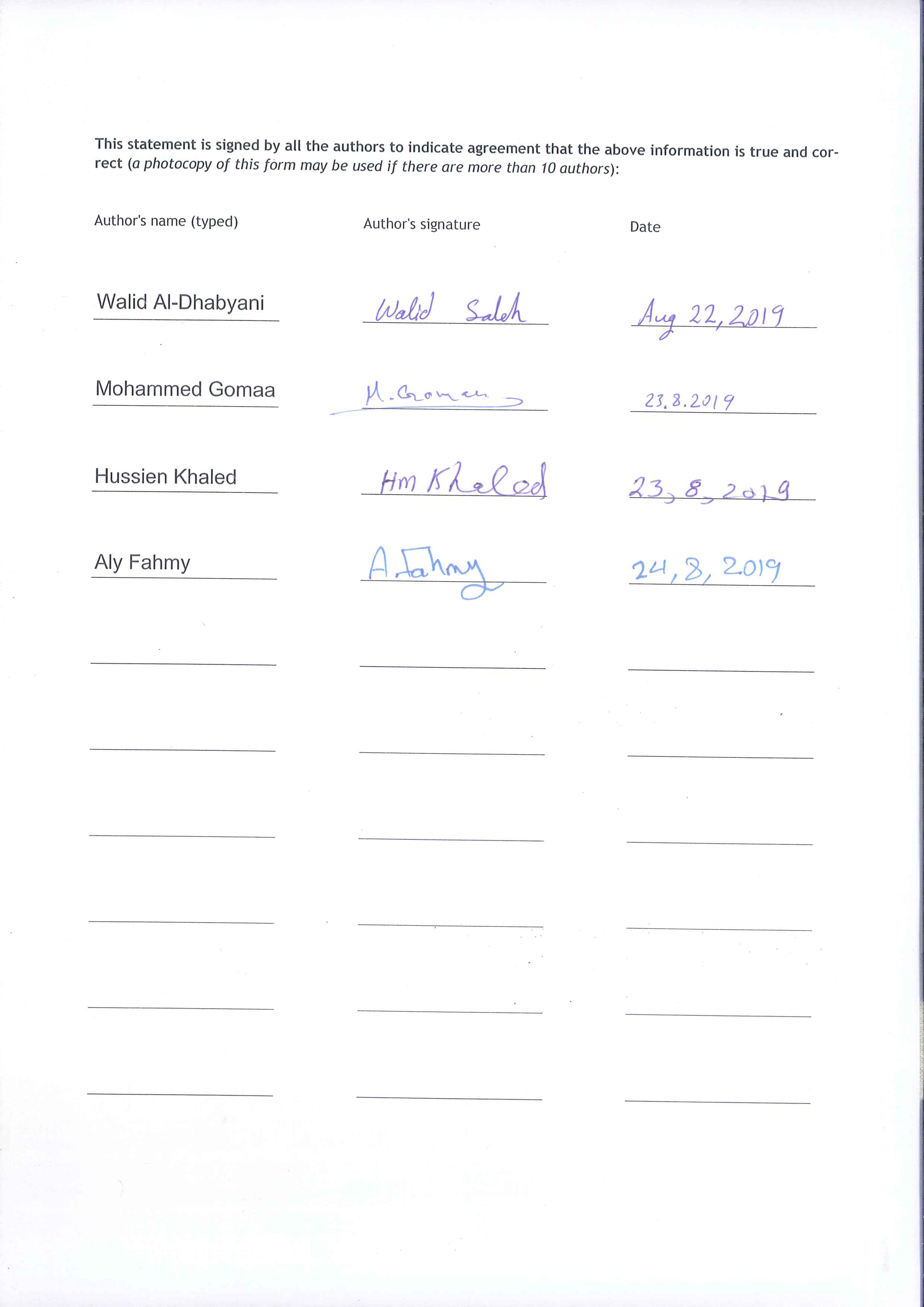

Supplement: Multimedia component 1 [file mmc1.zip › confilct_of_interset_statement_2.png]

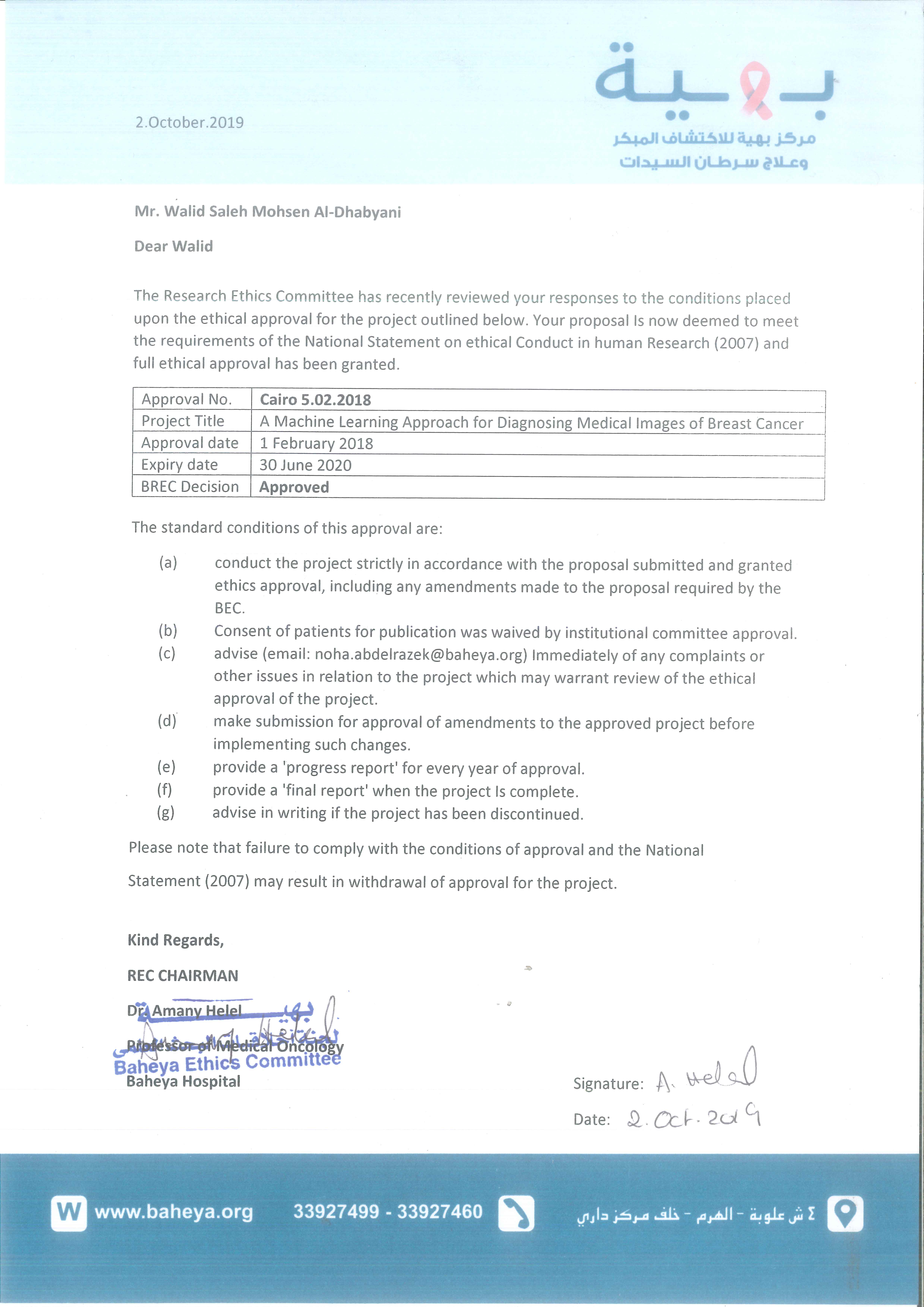

Supplement: Multimedia component 1 [file mmc1.zip › Ethical Committee V2.png]

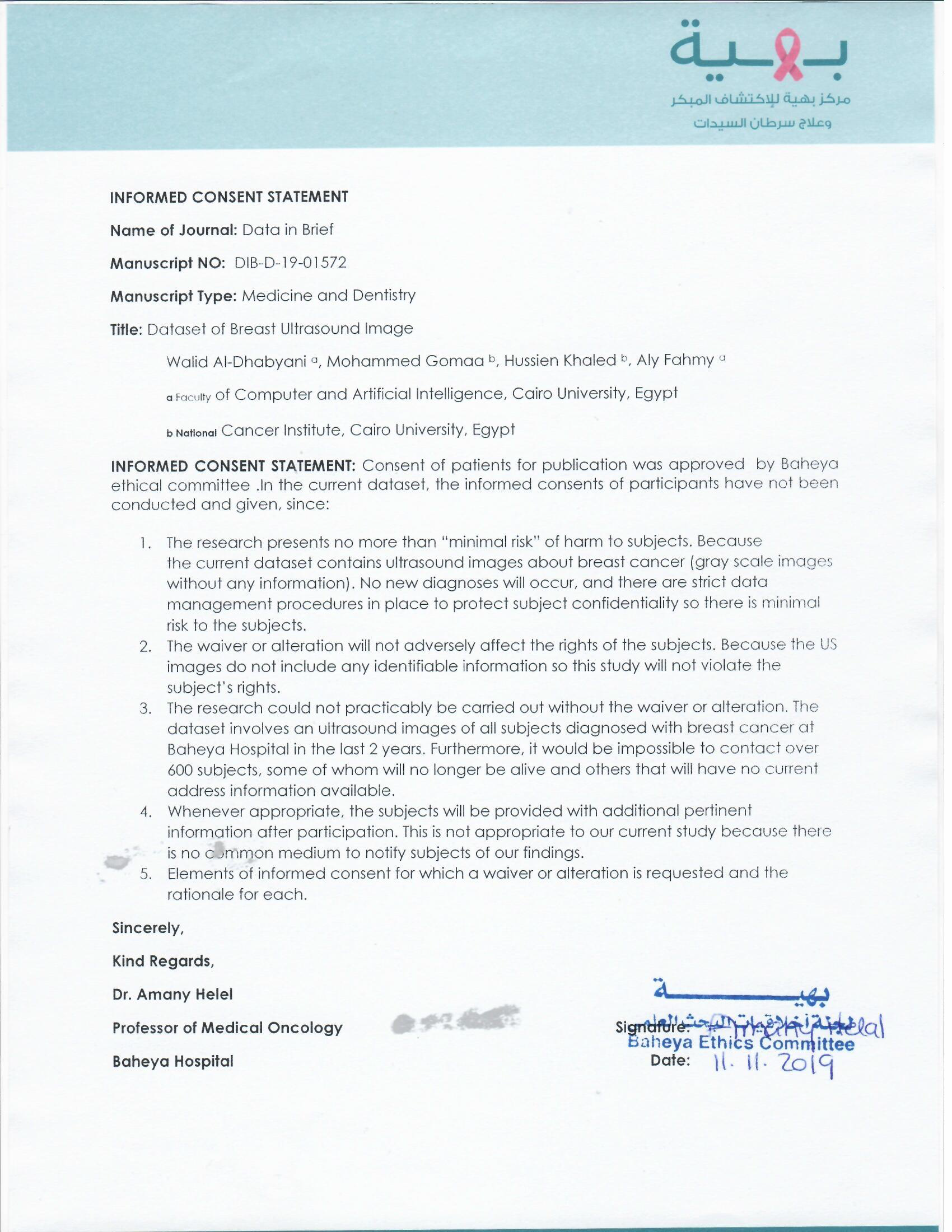

Supplement: Multimedia component 1 [file mmc1.zip › Informed consent statement.png]

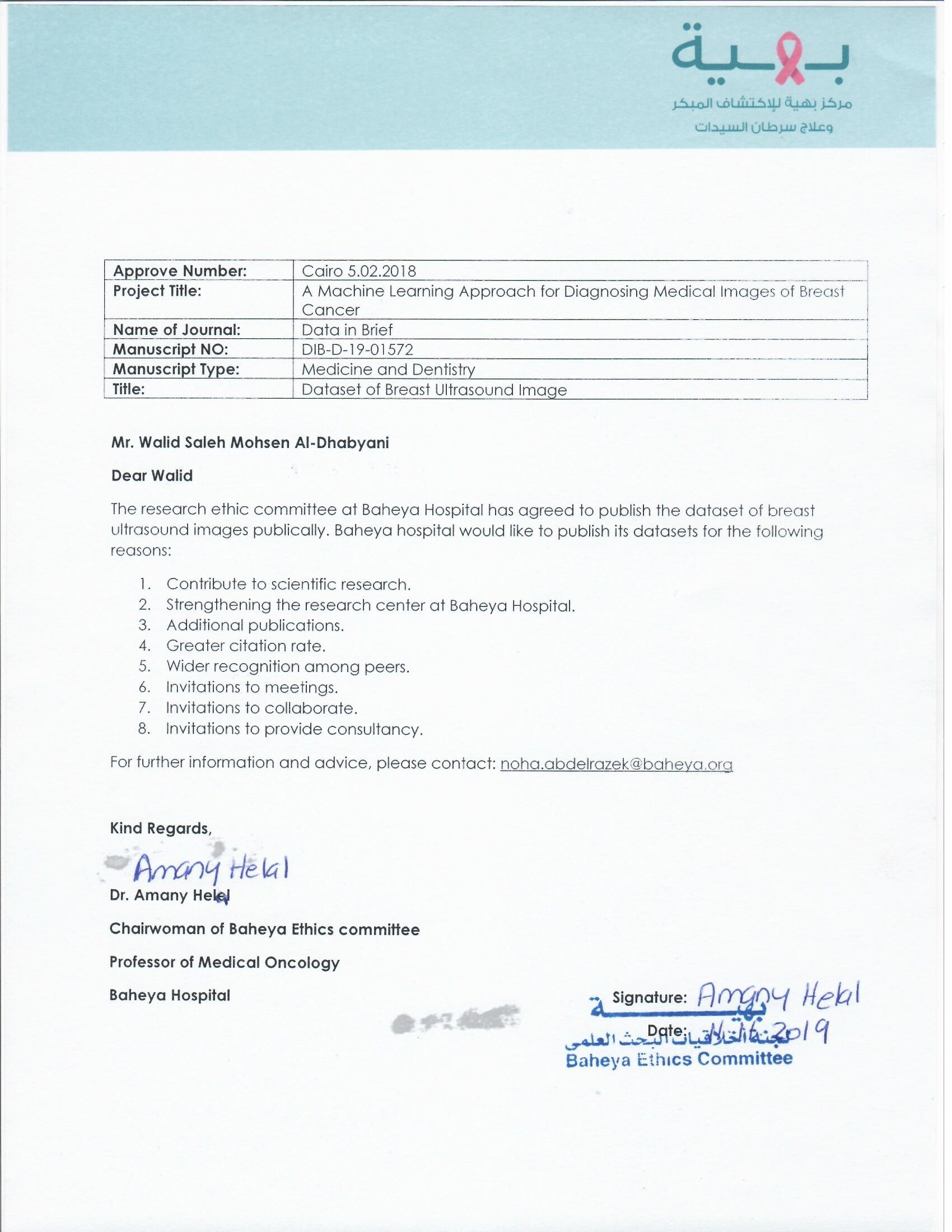

Supplement: Multimedia component 1 [file mmc1.zip › Baheya_publication_acceptance.png]
